# Supplementary figures and images for: Pharmacological inhibition of catalase induces peroxisome leakage and suppression of LPS induced inflammatory response in Raw 264.7 cell
Source: PLoS One. 2021 Feb 19;16(2):e0245799. doi: 10.1371/journal.pone.0245799 (PMC7894815; doi:10.1371/journal.pone.0245799)

**A**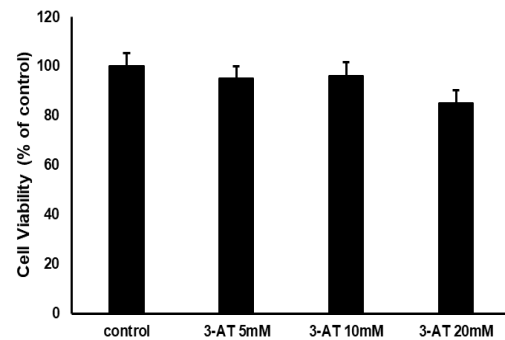**B**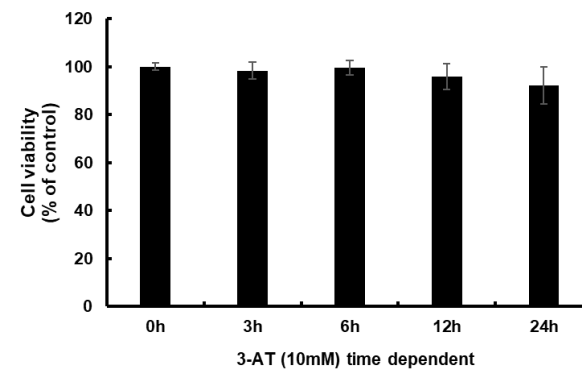**C**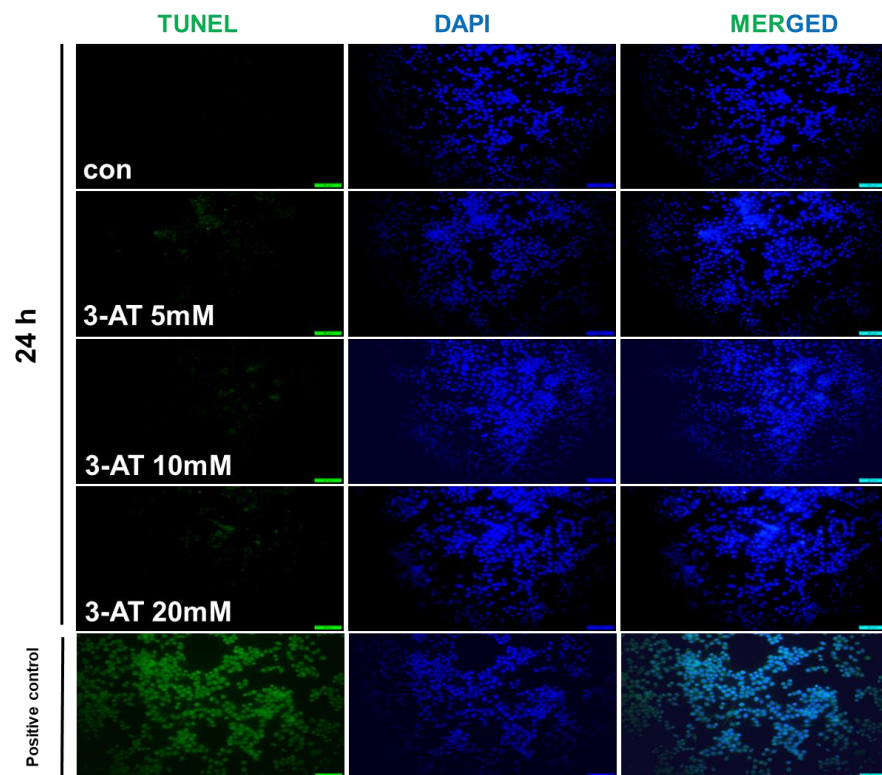**D**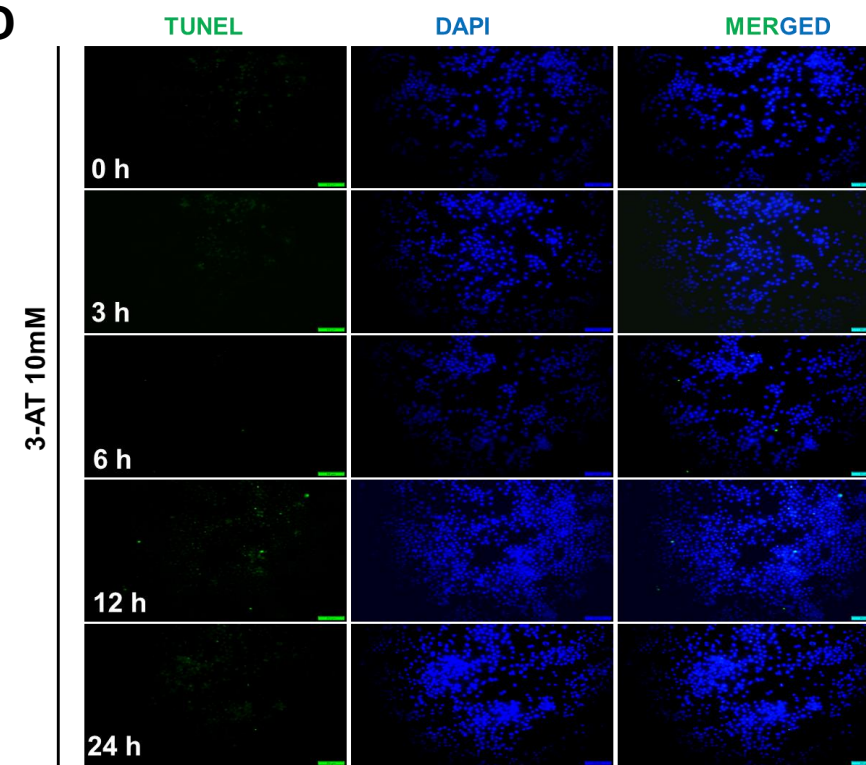

Supplement: S1 Fig — Cell viability was determined in (A) RAW264.7 cells treated with 3-AT at concentrations indicated in for 24 h and (B) 10 mM of 3-AT at time points indicated by MTT assay. (C-D) TUNEL assay obtained from RAW264.7 cells treated with 3-AT as in A and B. TUNEL-positive nuclei are indicated in green. Scale bar represents 50 μm. (PDF) [file pone.0245799.s001.pdf]

**A**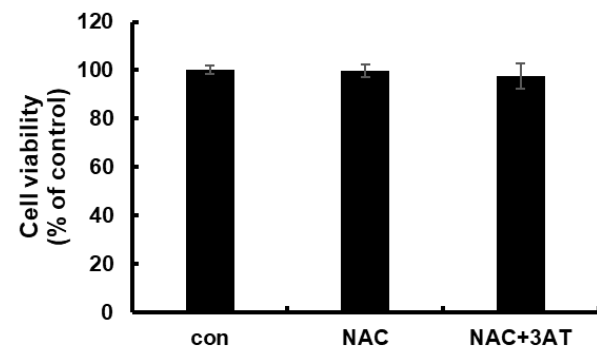**B**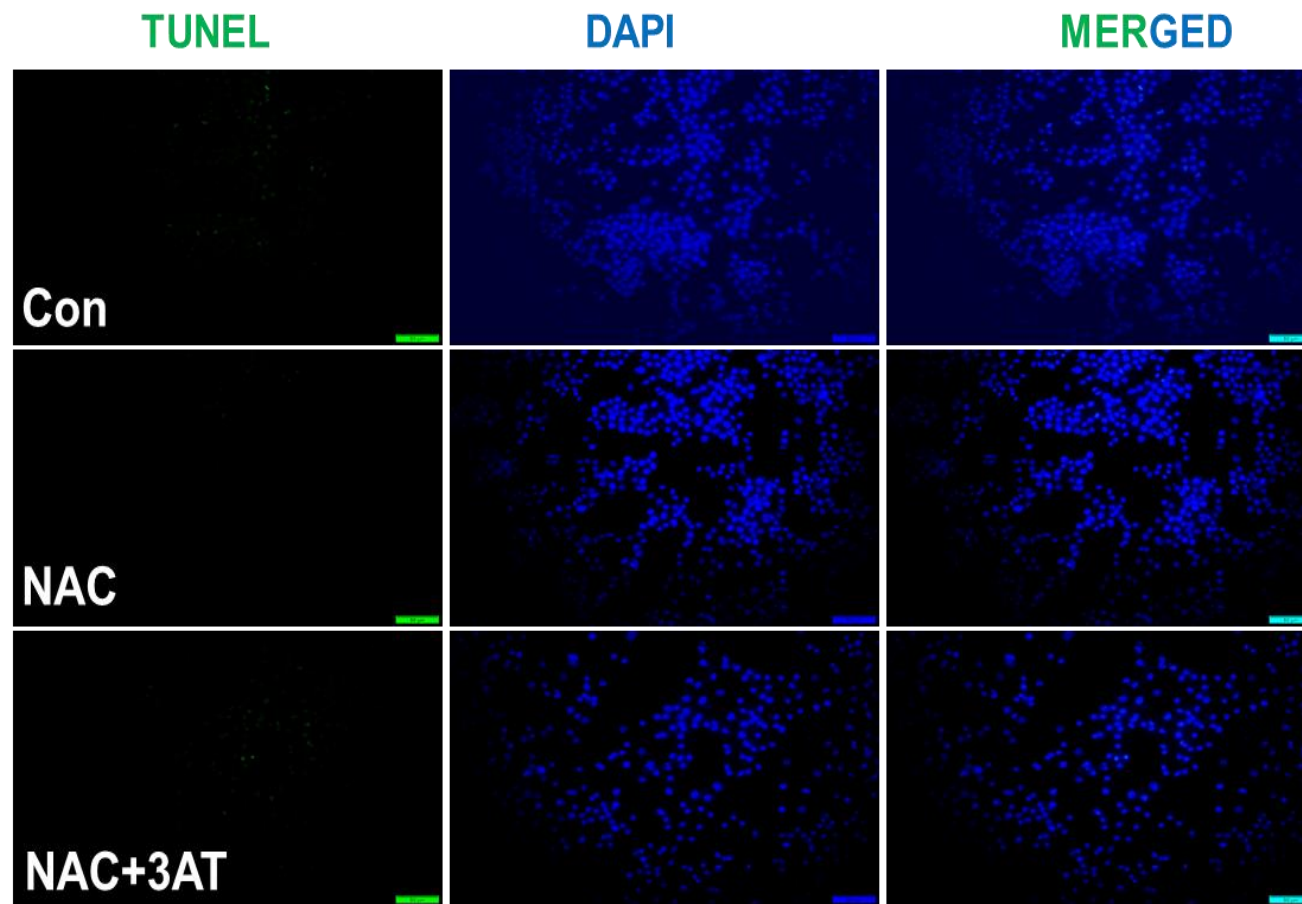

Supplement: S2 Fig — (A) Cell viability was measured by MTT assay in RAW264.7 cells treated with either NAC (2 mM) or co-treatment with NAC and 3-AT for 24 h. (B) TUNEL assay obtained from RAW264.7 cells treated A. Scale bar represents 50 μm. (PDF) [file pone.0245799.s002.pdf]

**A**

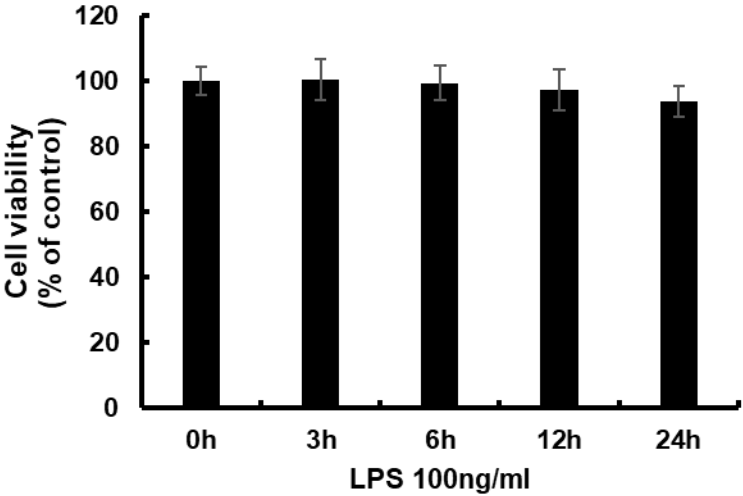

**B**

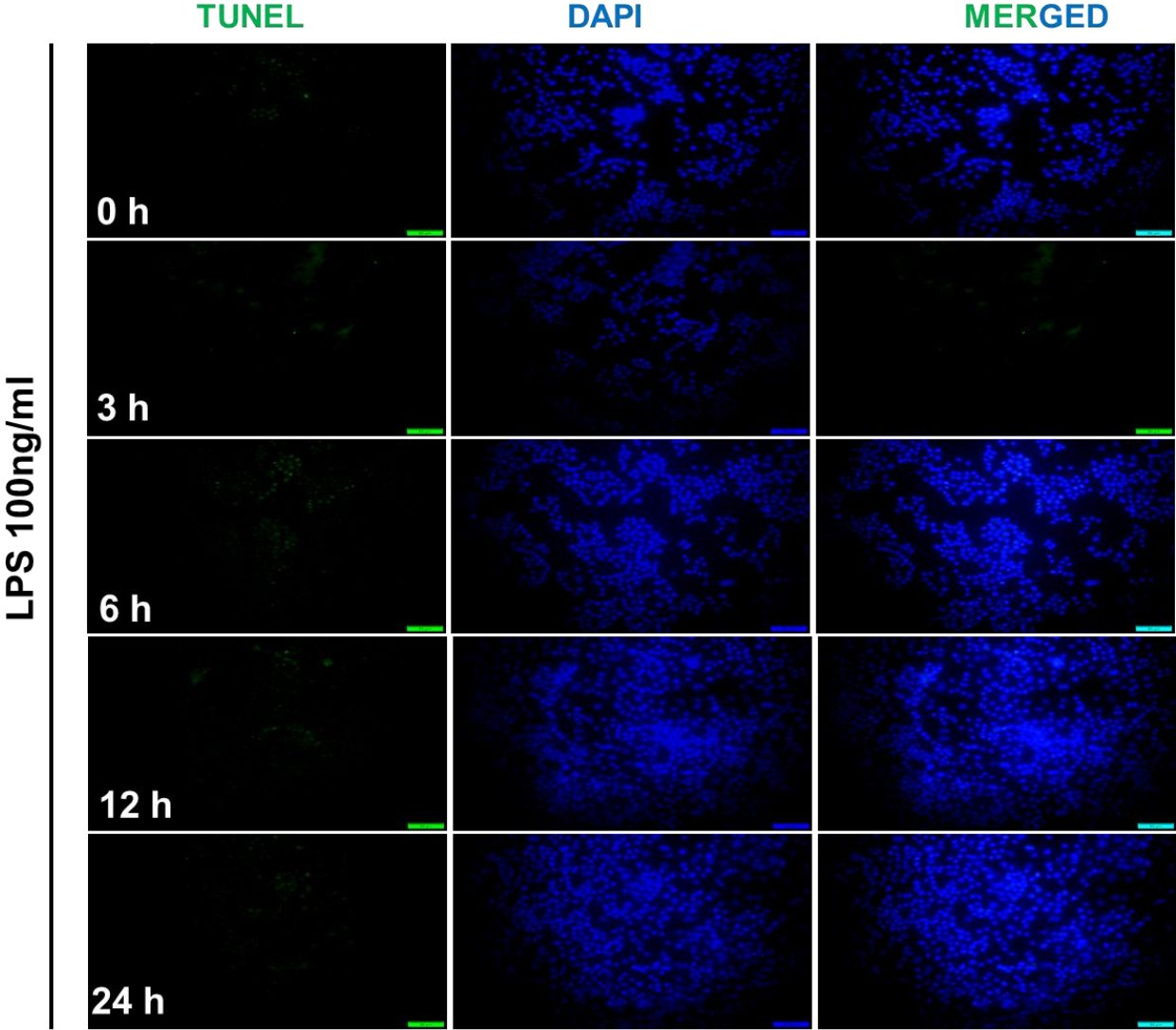

Supplement: S3 Fig — (A) Cell viability was measured by MTT assay in RAW264.7 cells treated with 100 ng/ml of LPS at time points indicated. (B) TUNEL assay obtained from RAW264.7 cells treated A. Scale bar represents 50 μm. (PDF) [file pone.0245799.s003.pdf]

**A**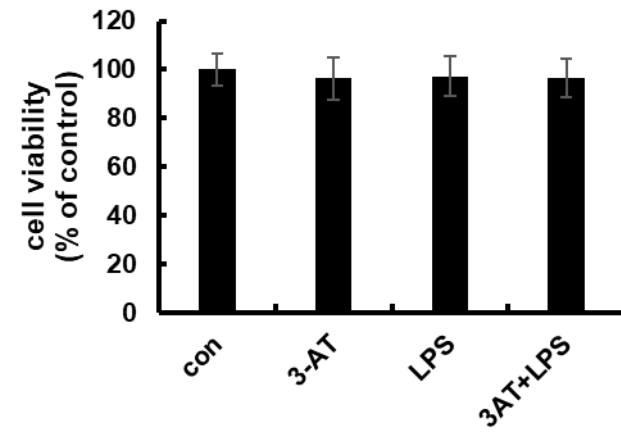**B**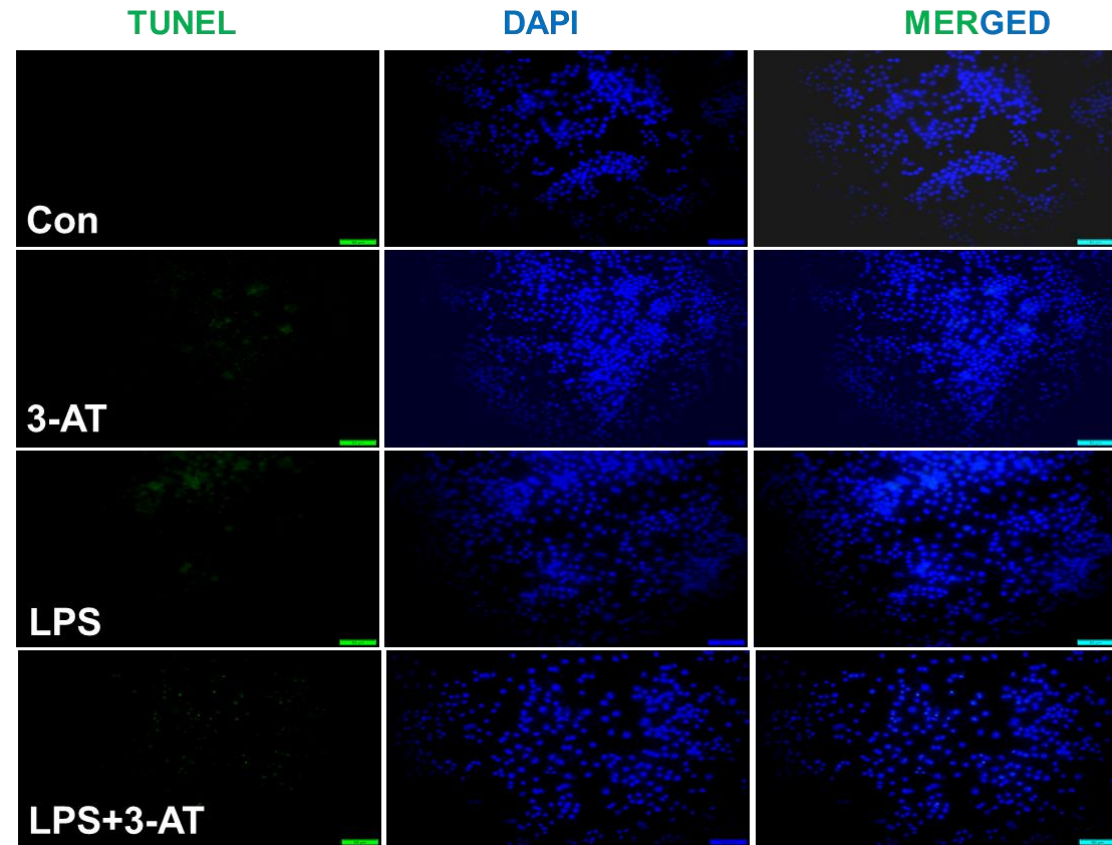

Supplement: S4 Fig — (A) Cell viability was measured by MTT assay in RAW264.7 cells treated with either 100ng/ml of LPS or 10 mM of 3-AT or both (LPS and 3AT) at time indicated. (B) TUNEL assay obtained from RAW264.7 cells treated A. Scale bar represents 50 μm. (PDF) [file pone.0245799.s004.pdf]

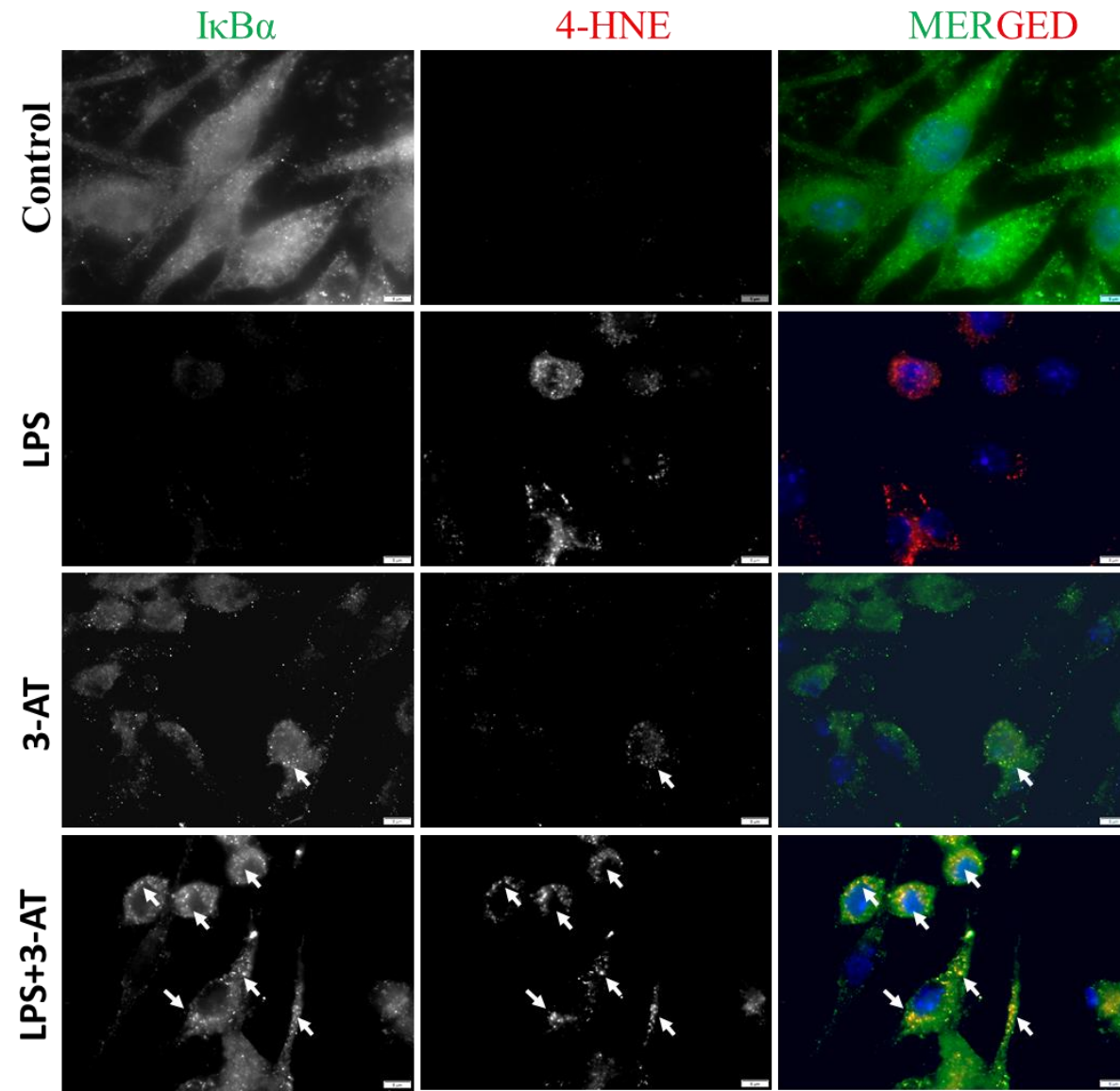

Supplement: S5 Fig — RAW 264.7 cells pre-treated with 10 mM of 3-AT for 12 h were further incubated with or without 100 ng/ml of LPS for additional 12 h. Cells were then subjected to immunofluorescence with anti-IκBα (green), anti-4-HNE (Red) and DAPI (blue). Arrow indicate the co-localization of IκBα with 4HNE (yellow). (PDF) [file pone.0245799.s005.pdf]

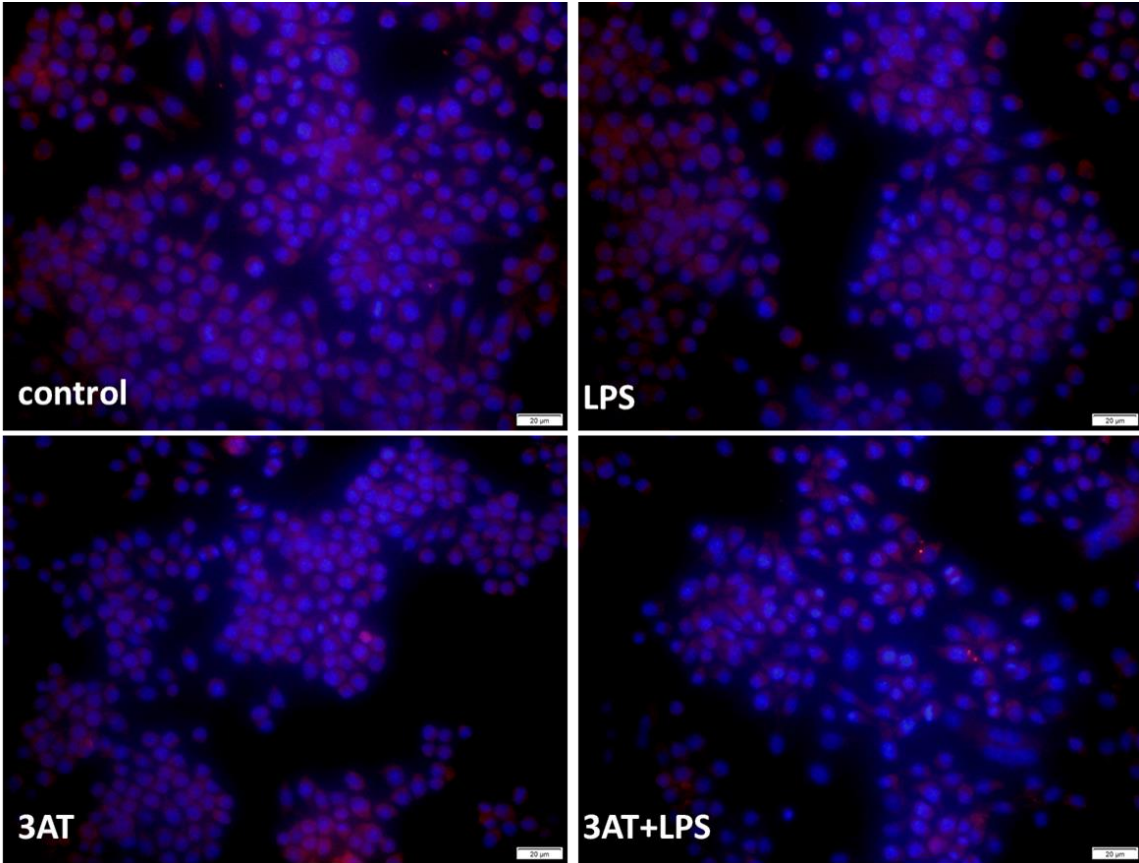

**Mitosox Red** **DAPI**

Supplement: S6 Fig — Representative red fluorescence image of MitoSOX Red from RAW 264.7 cells treated with 10 mM of 3-AT alone, 100 ng/ml of LPS alone, or combination of both for 24 h. Blue color represents DAPI stained. All images were processed and analyzed in a similar manner. (PDF) [file pone.0245799.s006.pdf]

**Figure 1B**

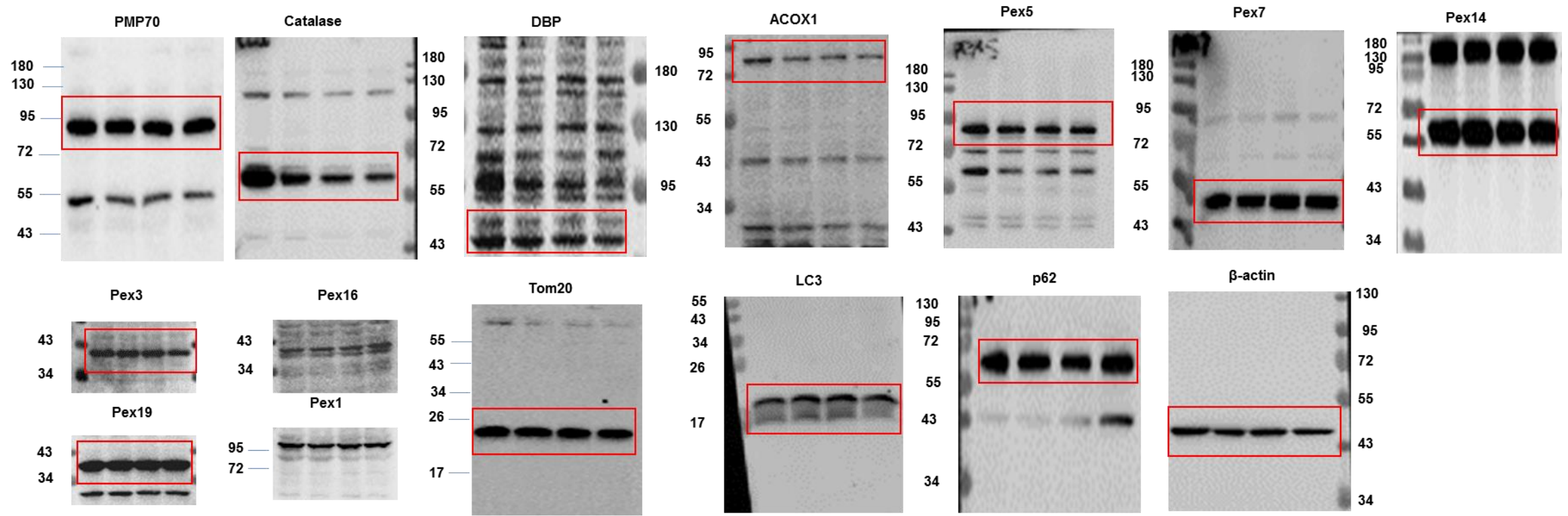

**Figure 1C**

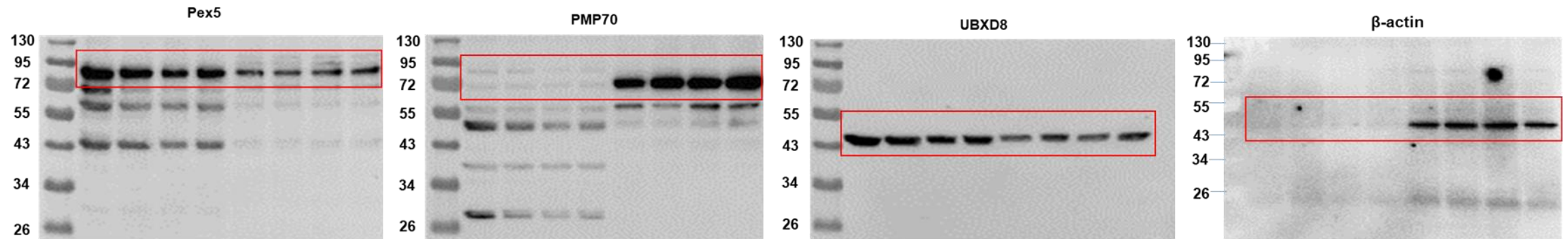

Supplement: S7 Fig — (PDF) [file pone.0245799.s007.pdf]

**Figure 2F**

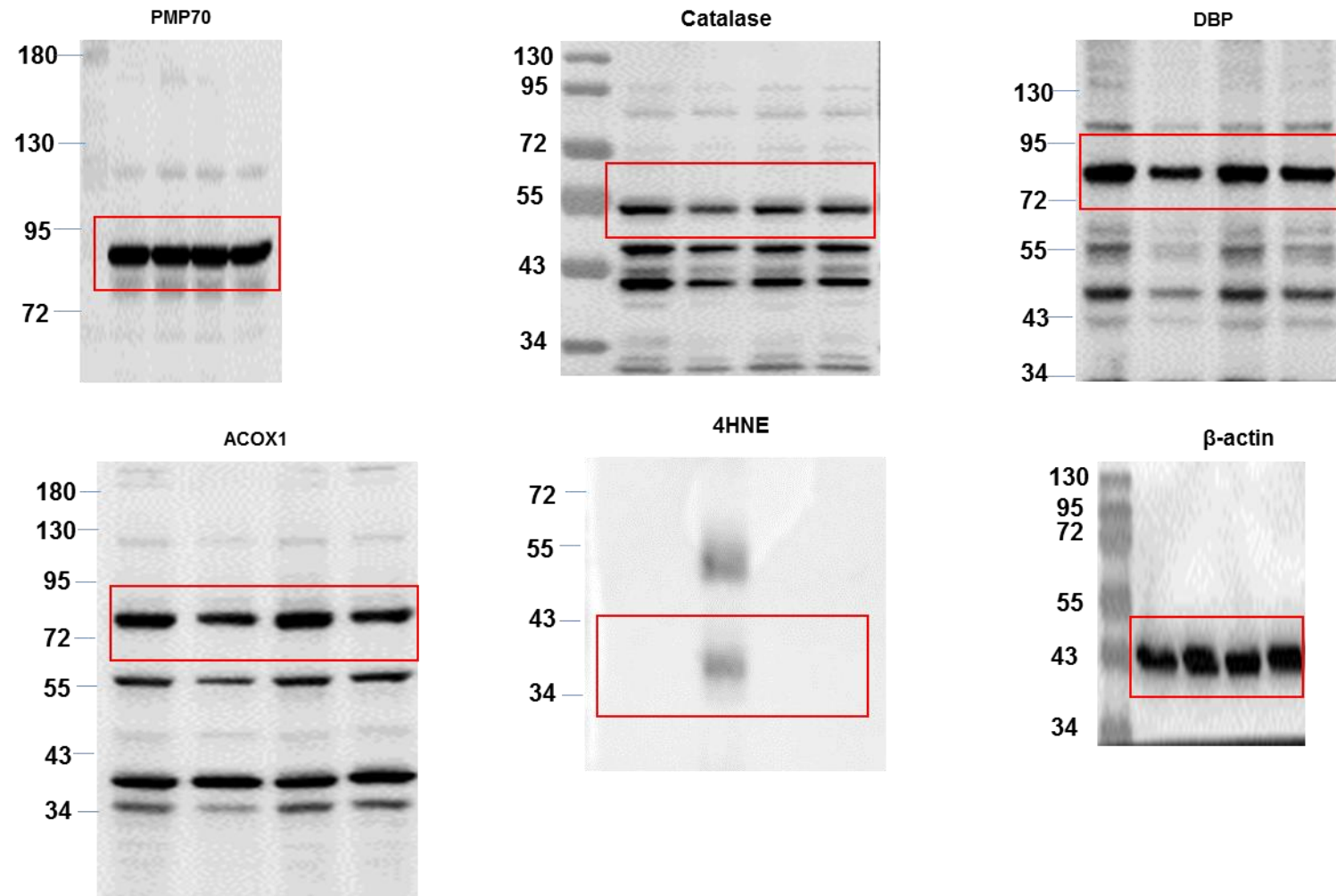

Supplement: S8 Fig — (PDF) [file pone.0245799.s008.pdf]

### Figure 3A

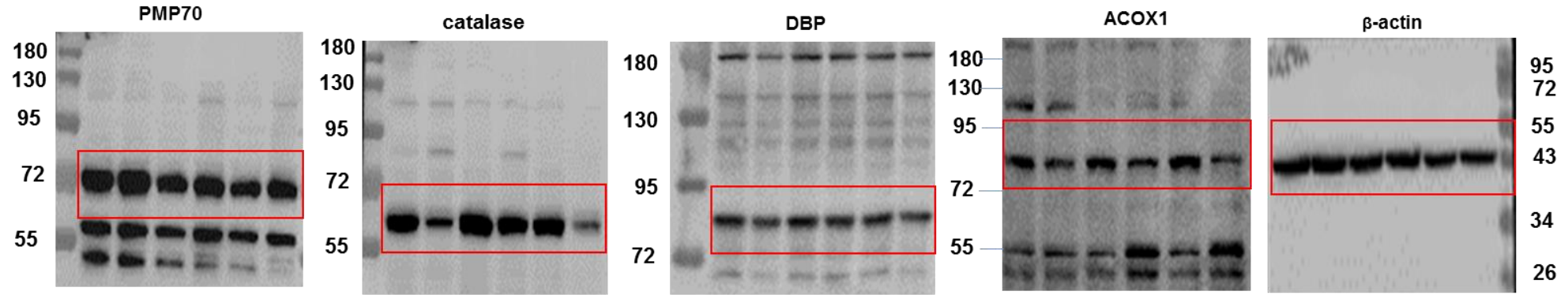

### Figure 3B

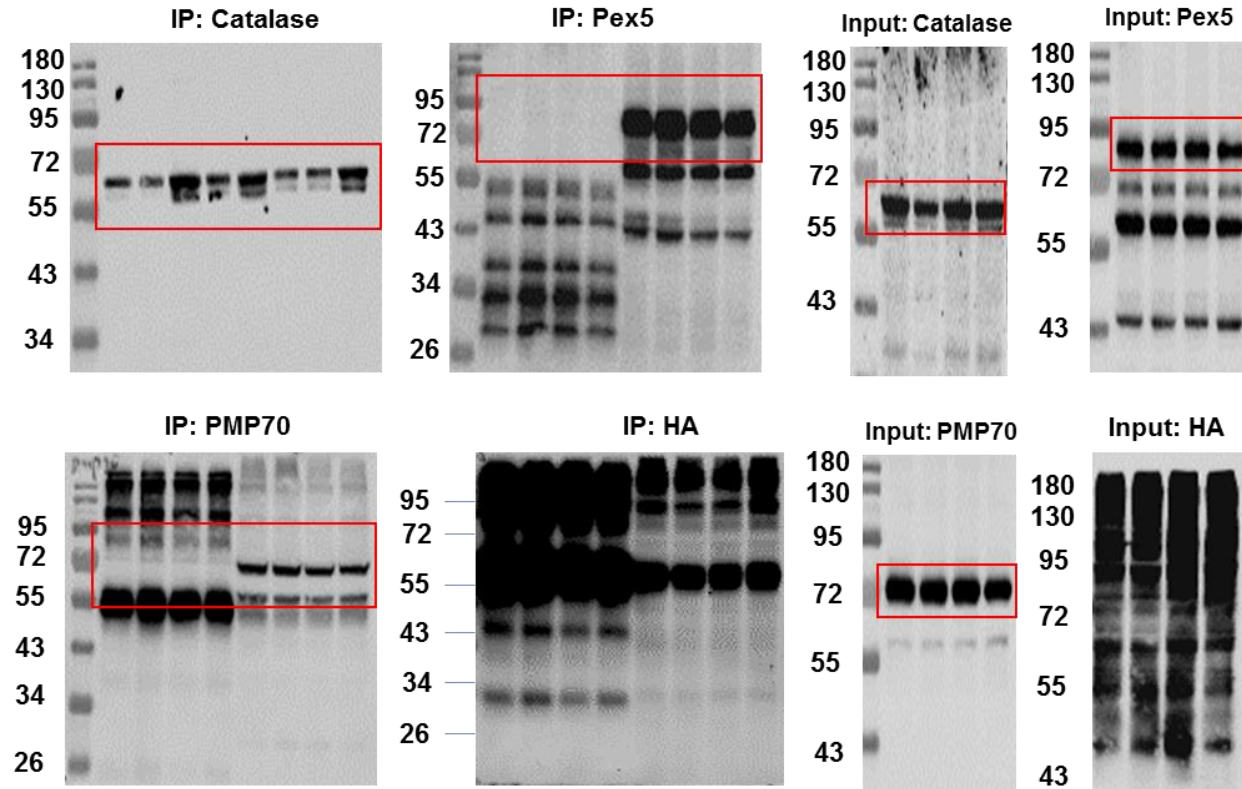

### Figure 3D

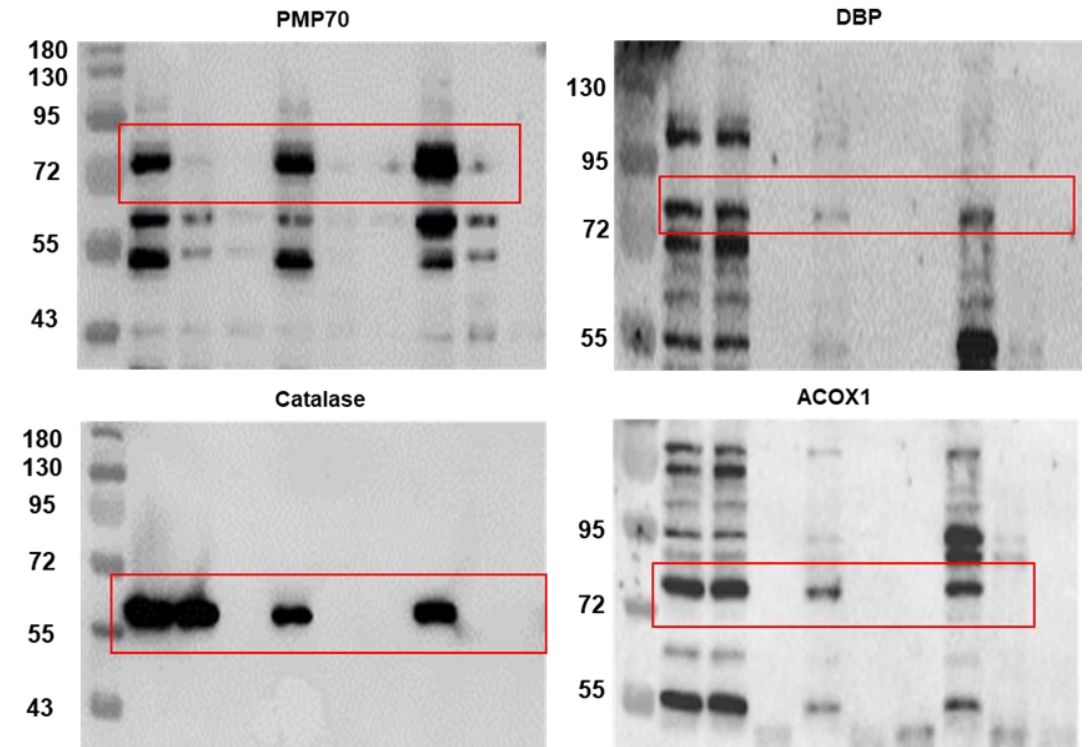

Supplement: S9 Fig — (PDF) [file pone.0245799.s009.pdf]

**Figure 4E**

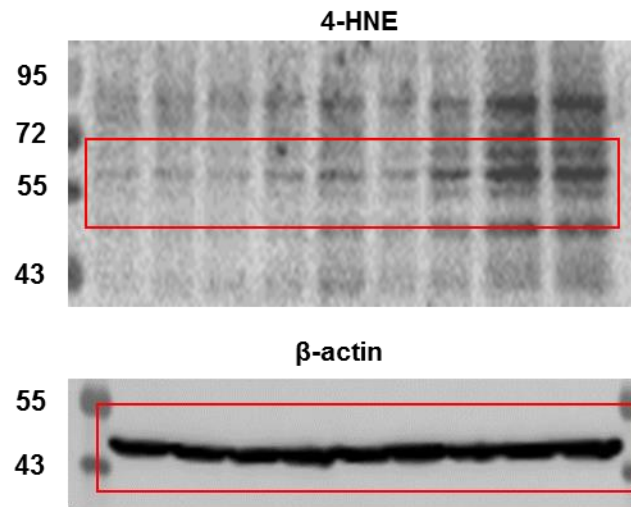

**Figure 4F**

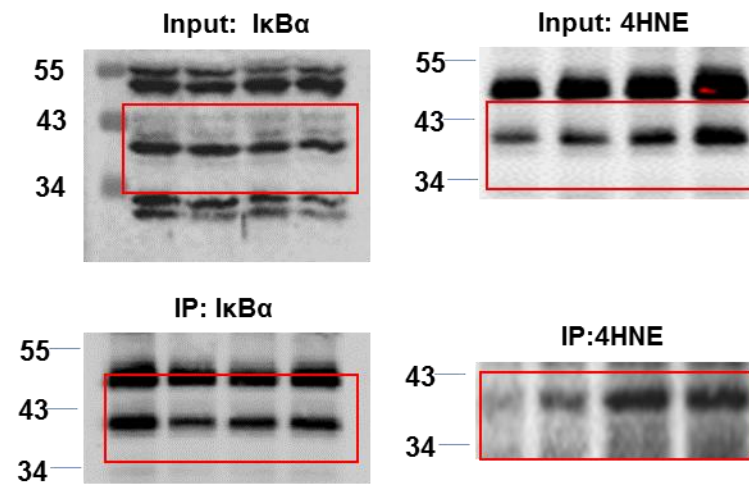

**Figure 4G**

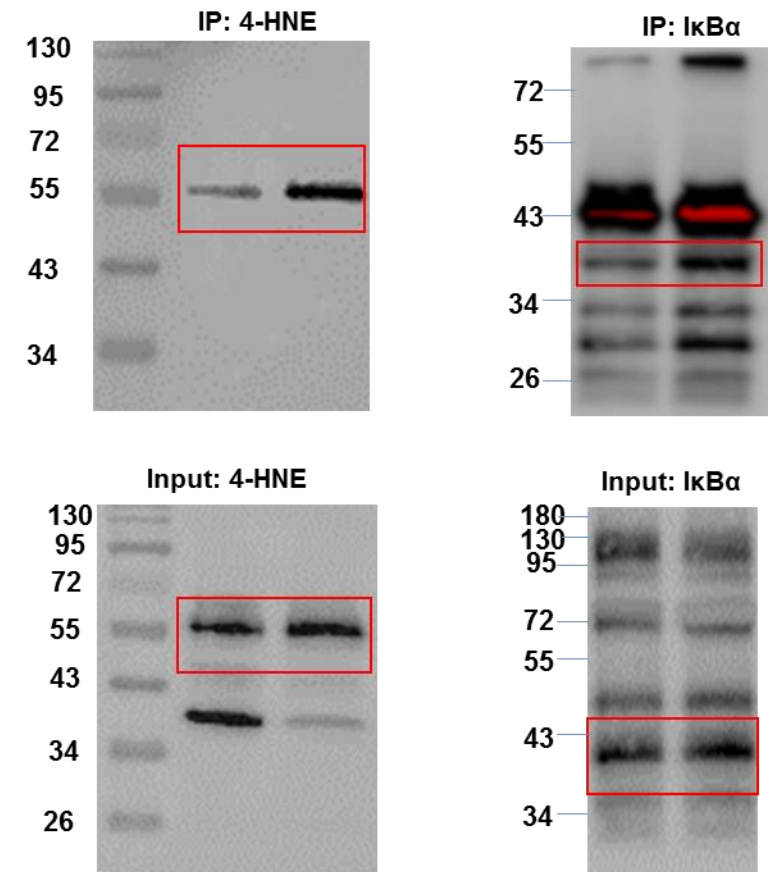

Supplement: S10 Fig — (PDF) [file pone.0245799.s010.pdf]

**Figure 5A**

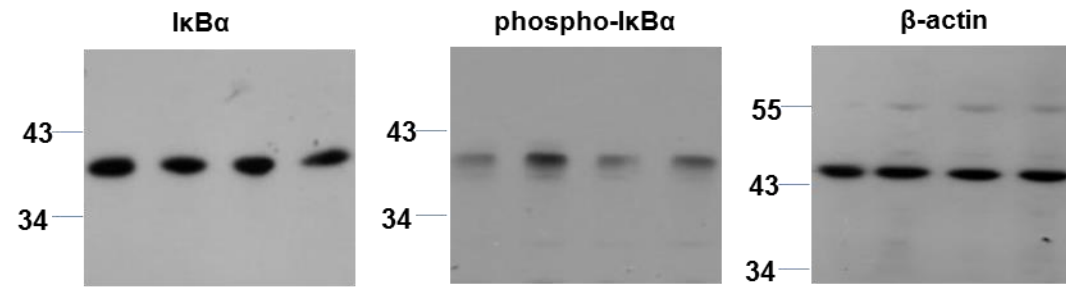

**Figure 5B**

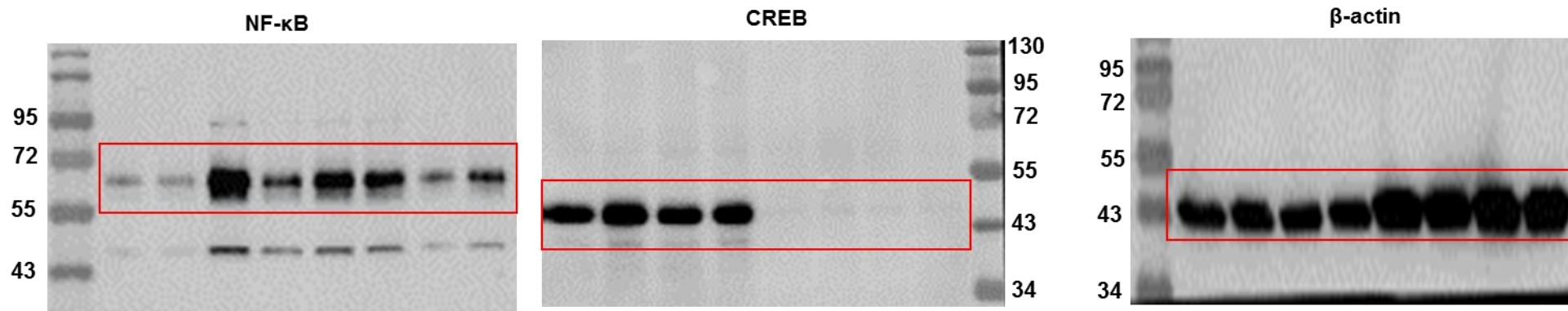

Supplement: S11 Fig — (PDF) [file pone.0245799.s011.pdf]
