# Supplementary material for: Pharmacological inhibition of catalase induces peroxisome leakage and suppression of LPS induced inflammatory response in Raw 264.7 cell
Source: PLoS One. 2021 Feb 19;16(2):e0245799. doi: 10.1371/journal.pone.0245799 (PMC7894815; doi:10.1371/journal.pone.0245799)
Supplement: Attachment — Submitted filename: response to reviewers.docx [file pone.0245799.s012.docx]

**Response to Reviewers**

**Our response**

**We thank PLOS ONE editorial team for their valuable comments.**

Journal Requirements:

When submitting your revision, we need you to address these additional requirements.

**1. Please ensure that your manuscript meets PLOS ONE's style requirements, including those for file naming. The PLOS ONE style templates can be found at**

https://journals.plos.org/plosone/s/file?id=wjVg/PLOSOne_formatting_sample_main_body.pdf and

https://journals.plos.org/plosone/s/file?id=ba62/PLOSOne_formatting_sample_title_authors_affiliations.pdf

**Response: We have modified the present manuscript according to the PLOS ONE's style requirements**

**2. We note that you have included the phrase “data not shown” in your manuscript. Unfortunately, this does not meet our data sharing requirements. PLOS does not permit references to inaccessible data. We require that authors provide all relevant data within the paper, Supporting Information files, or in an acceptable, public repository. Please add a citation to support this phrase or upload the data that corresponds with these findings to a stable repository (such as Figshare or Dryad) and provide and URLs, DOIs, or accession numbers that may be used to access these data. Or, if the data are not a core part of the research being presented in your study, we ask that you remove the phrase that refers to these data.**

**Response: We replaced the “data not shown” in manuscript with cell viability for different doses of 3-AT shown as a supplementary figure (S1 A-B).**

**3. Please include captions for your Supporting Information files at the end of your manuscript, and update any in-text citations to match accordingly. Please see our Supporting Information guidelines for more information:** [**http://journals.plos.org/plosone/s/supporting-information**](http://journals.plos.org/plosone/s/supporting-information)**.**

**Response: We added the caption for the Supporting Information files at the end of manuscript and in-text citations are matched according to PLOS ONE's style requirements**

**4.PLOS ONE now requires that authors provide the original uncropped and unadjusted images underlying all blot or gel results reported in a submission’s figures or Supporting Information files. This policy and the journal’s other requirements for blot/gel reporting and figure preparation are described in detail at https://journals.plos.org/plosone/s/figures#loc-blot-and-gel-reporting-requirements and https://journals.plos.org/plosone/s/figures#loc-preparing-figures-from-image-files. When you submit your revised manuscript, please ensure that your figures adhere fully to these guidelines and provide the original underlying images for all blot or gel data reported in your submission. See the following link for instructions on providing the original image data:** [**https://journals.plos.org/plosone/s/figures#loc-original-images-for-blots-and-gels**](https://journals.plos.org/plosone/s/figures#loc-original-images-for-blots-and-gels).

**Response: All the uncropped western blot data in the result section have already been provided in the original version of this manuscript as Supplemantary images and gel (at the end of the manuscript)**

**In your cover letter, please note whether your blot/gel image data are in Supporting Information or posted at a public data repository, provide the repository URL if relevant, and provide specific details as to which raw blot/gel images, if any, are not available. Email us at plosone@plos.org if you have any questions.**

**Response: Uncropped western blot data with specific details have been submitted as supporting information (uncropped western blot for Figure 1-5)**

5. Review Comments to the Author

Please use the space provided to explain your answers to the questions above. You may also include additional comments for the author, including concerns about dual publication, research ethics, or publication ethics. (Please upload your review as an attachment if it exceeds 20,000 characters)

**Reviewer #1: The article “Pharmacological inhibition of catalase induces peroxisome leakage and suppression of LPS induced inflammatory response in Raw246.7 cell” by Mu et al report the role of catalase and effects of peroxisome derived ROS on lipopolysaccharide (LPS) mediated inflammatory pathway are largely unknown. They showed that inhibition of catalase by 3-aminotriazole (3-AT) results in the generation of peroxisomal ROS, that leads to the leakage of peroxisomal matrix proteins into the cytoplasm in RAW246.7 cells. Furthermore, the author showed that 3-AT promotes the formation of 4HNE-IκBα adduct which directly interferes with the LPS induced NF-κB activation. The paper is interestingly and deserve publication alsthou few points should be addressed:**

**Major revision
1. The survival of the cells under each chemical treatment at each time point and under the selected concentrations should be shown (apoptosis assay or Cas3 staining).**

**Response: To address the requirement of cell survival under chemical treatments, we have included 3-(4,5-dimethylthiazol-2-yl)-2,5-diphenyltetrazolium bromide (MTT) assay as well as Terminal deoxynucleotidyl transferase dUTP nick-end labeling (TUNEL) assay shown as Supplementary figures (S1, S2, S3 and S4)**

**2. The interaction between 4HNE and IκBα should be shown also by immunofluorescence**

**Response: We highly appreciate the reviewer's concern regarding the inclusion of additional immunofluorescence data for showing 4HNE and IκBα interaction. Although, 4HNE-protein interaction has been recently demonstrated through indirect immunofluoresence and proximity ligation assay [1], we believe that immunoprecipitation (IP) is the standard method to study the interaction of 4HNE with other proteins [1, 2] and immunofluorescence method could be just an adjunct to standard IP. Furthermore, we have shown that 4HNE- IκBα interaction through the immunoprecipiation reaction using both anti-IκBα (Fig 4F) and anti 4HNE (Fig 4G). These immunoprecipitation data clearly suggest the existence of interaction between 4HNE and IκBα in the presence of 3-AT thereby negating the requirement for additional method for showing 4HNE-IκBα interaction.**

**3. The methods and software used to quantify pictures and blots should be described in details in the methods**

**Response: In the revised manuscript we have included all the necessary methods and software used to quantify fluorescent images and blots in Materials and Methods and figure legend where applicable**

**1. Ratio of catalase positive peroxisomes to total peroxisomes per cell (Figure 1F). Catalase and PMP70 puncta were counted from cells labelled with anti-PMP7O and anti-catalase. Ratio of catalase positive peroxisome was calculated by dividing total number of catalase puncta to the total number of PMP70 puncta per cell. At least 30 cells were counted in each experiment.**

**2. Measurement of DCFDA intensity**

**Quantification of DCFDA intensity was carried out as previously reported [3, 4], with slight modification. Cells were grown in coverslips. After treating cells with desired drugs at indicate time points, DCFDA was added to culture medium at a final concentration of 10** [**µM**](http://acronymsandslang.com/definition/7820032/%C2%B5M-meaning.html#:~:text=What%20is%20the%20abbreviation%20for%20micromolar%20%28micromoles%2Fliter%29%3F%20micromolar,in%20Common%20%2F%20Miscellaneous%20%2F%20Community%20by%20AcronymsAndSlang.com) **and incubated for 30 min at 37^o^C. Cells were then washed three times with PBS. To detect the nuclei, coverslips were mounted on slides with the Prolong Gold antifade reagent containing DAPI (#P36931, Sigma) and examined under fluorescence microscope (IX71, Olympus, Tokyo, Japan). DCFDA intensity was measured from full image with similar cell distribution in each condition by Image J software. Intensities from at least 10 full images were measured per condition for each experiment. The results were interpreted as arbitrary unit or fold of control. We added above sentences in Material and Methods.**

**3. Measurement of HyPer SKL intensity**

**Area around the HyPer-SKL fluorescent signal was considered as a region of interest for the measurement of intensity by Image J software. Intensity from at least 30 HyPer-SKL transfected cells was measured per condition for each experiment. The results were interpreted as a fold of control. We added above sentence in Material and Methods.**

**4. Quantification of western blot band.**

**Densitometric analysis of western blot bands was measured by Image J Software. The quantification of bands reflects the relative amounts as a ratio of each protein band relative to the lane’s loading control (β-actin). We added above sentence in Material and Methods.**

**Minor revision
1. The English overall the text needs improvement. Mainly in the abstract, introduction and discussion.**

**Response: We have now carefully corrected all the grammatical errors as well as sentence rearrangement for making our manuscript more coherent.**

**2. In the abstract define HNE**

**Response: In the revised manuscript we have now added 4HNE (4-hydroxy-2-nonenal) description and full form**

**3. The reference Di Cara et al, Immunity 2017 should be cited in discussion since in this paper it was demonstrated for the first time the role of peroxisomes the role of peroxisomes redox and peroxisomal Catalase in mediating the regulation of NF-kβ transcriptional activity in macrophages of Drosophila melanogaster.**

**Response: We agree with the reviewer and we have cited this paper in the revised manuscript.**

**Also the reference below is not correctly reported the first author is:**

**Di Cara F Andreoletti P, Trompier D, Vejux A, Bülow MH, Sellin J, et al. Peroxisomes in
Immune Response and Inflammation. Int J Mol Sci. 2019;20(16):3877. pmid: 31398943**

**Cara FD, Andreoletti P, Trompier D, Vejux A, Bülow MH, Sellin J, et al. Peroxisomes in
Immune Response and Inflammation. Int J Mol Sci. 2019;20(16):3877. pmid: 31398943**

**Response: In the revised manuscript we have now corrected this reference**

**References:**

1. Mihalas BP, De Iuliis GN, Redgrove KA, McLaughlin EA, Nixon B. The lipid peroxidation product 4-hydroxynonenal contributes to oxidative stress-mediated deterioration of the ageing oocyte. Sci Rep. 2017;7(1):6247. PMID: 28740075.
2. Dou X, Li S, Wang Z, et al. Inhibition of NF-κB activation by 4-hydroxynonenal contributes to liver injury in a mouse model of alcoholic liver disease. Am J Pathol. 2012;181(5):1702-1710. PMID: 22982442.
3. Surma M, Handy C, Chang J, Kapur R, Wei L, Shi J. ROCK1 deficiency enhances protective effects of antioxidants against apoptosis and cell detachment. PLoS One. 2014;9(3):e90758PMID: 24595357
4. Shen WJ, Hsieh CY, Chen CL, Yang KC, Ma CT, Choi PC, Lin CF. A modified fixed staining method for the simultaneous measurement of reactive oxygen species and oxidative responses. Biochem Biophys Res Commun. 2013;430(1):442-7. PMID: 23178299.

**Reviewer #2: This paper by Mu, Maharjan and Dutta et al., concerns the ability for catalase inhibition to suppress inflammatory responses in the macrophage cell line RAW 264.7. The authors found that 3-AT induced leakiness in peroxisomes, resulting in the production of peroxisomal ROS, 4HNE production and subsequent inhibition of NFkB signaling by adduct formation in the NFkB pathway. The evidence comes from conventional molecular and cell biological assays and is carefully performed, reported and interpreted. This study will be of interest to scientists studying reactive oxygen signaling, peroxisome biology and inflammation. I have some comments which the authors are encouraged to address:**

**Response: We thank this reviewer for valuable suggestions and positive criticisms.

• The authors suggest that lipid peroxidation is not dependent on processes that are induced by LPS treatments or the associated ROS. The authors are encouraged to discuss or provide additional experimental support regarding how their results in Figure 4 differ from previously published work by Yang et al., J Neuroinflammation 2018, which found that LPS did indeed induce 4-HNE production in microglial cells (doi: 10.1186/s12974-018-1232-3). See also, Choi et al J Neuroscience 2007 (doi: 10.1523/JNEUROSCI.5417-06.2007) and Chen et al Frontiers in Immunology 2019 (doi: 10.3389/fimmu.2019.01904) – this last one used bone marrow-derived macrophages and thus should be given particular note as it is unlikely simply because of the differences between macrophages and microglia (which are already considered quite similar in function).**

**Response: We thank this reviewer for allowing us to discuss the discrepancies observed between our study and earlier reports regarding the role of LPS on ROS generation and 4HNE production.**

**We have shown that LPS (100 ng/ml) does not increase global ROS generation until 12 h (Fig 4C). Although, global ROS generation was evident at 24 h of LPS treatment, 4HNE production was not evident at the same time point (Fig 4E). Furthermore, transcriptional upregulation of pro-inflammatory cytokine genes was found to reach maximum at 12 h (Fig 4A) whereas pro-inflammatory cytokines in the cultured medium was increased at 24 h of LPS treatment. These data suggest that global ROS was not involved in the transcriptional activation of pro-inflammatory cytokine genes by LPS. Rather, ROS production observed at 24 h of LPS treatment could be due to the effect of release of pro-inflammatory cytokines in the medium.**

**Our data showed that LPS does not involve in global ROS generation and 4HNE production is in contrast with those reported by Yang et al., [5]. The observed discrepancies between two studies could be due to the differences in the experimental conditions. In the report by Yang et al., BV-2 microglial cells were cultured in 5% serum medium, serum-starved for 3 h, and followed by LPS (100 ng/ml) treatment in 2% free fatty acids free BSA medium. Therefore, an increase in ROS and 4HNE production caused by LPS (100 ng/ml) as shown by Yang et al., could be due to the use of serum-free medium or low serum constituents in culture medium which might have triggered robust ROS generation and 4HNE production. As serum-starvation could trigger ROS generation in wide variety of cells [6], LPS treatment in low serum constituent might boost ROS generation and increase lipid peroxidation thereby releases 4HNE.**

**Similarly, the discrepancy observed between our study and those by Chen et al., [7] in terms of 4HNE production could be due to the use of different concentrations of LPS and treatment strategy. We added 100 ng/ml LPS in cultured medium whereas Chen et al., have used 1000 ng/ml LPS in primary bone marrow derived macrophage (BMDM) to measure 4HNE production. It is surprising to see that Chen et al., could observe the robust 4HNE production without affecting the release of pro-inflammatory cytokines by LPS. In addition Choi et al., have demonstrated that LPS injection in rat triggers 4HNE production in striatum. Since striatum is a complex tissue composed of neuronal cluster and other cell types including microglial, it is not certain whether LPS injection triggered 4HNE generation is actually derived from microglial cells in the striatum. We added this content in Discussion**

**• The authors focused only on whole-cell ROS production and peroxisome-specific ROS production, but did not give any attention to mitochondrial ROS. mROS is known to be significantly induced by LPS treatment, participates in lipid peroxidation, and has been shown to be induced by 3-AT treatment in vitro (Walton and Pizzitelli, Frontiers in Physiology 2012, doi: 10.3389/fphys.2012.00108). Mitochondria are one of the most potent contributors of ROS to the cell, and supporting evidence on the role of mitochondrial ROS in this process would provide significant strength to this study.**

**Response: We have shown that LPS (100 ng/ml) increased global ROS generation at 24 h (Fig 4C) when the transcriptional activation of pro-inflammatory cytokine genes has been subsided (Fig 4A). Since mitochondria are an one of key organelles of ROS production, it is reasonable to speculate that mitochondria play a crucial role in contribution of global ROS generation at 24 h in the presence of LPS. Although, global ROS level was increased at 24 h, lipid peroxidation was not evident at the same time point (Fig 4E), suggesting that amount of global ROS generated might not be sufficient to induce lipid peroxidation in our study. As describe in the earlier comment, induction of lipid peroxidation by LPS observed in the previous report [5, 7, 8] could merely due to the differences in experimental conditions, including dose of LPS, and method of administration of LPS, which could enhance the mitochondrial ROS production leading to lipid peroxidation. Because we have found that 3-AT prevents the LPS mediated transcriptional activation of pro-inflammatory cytokine genes independent with global ROS status, further assessment for the effect of 3-AT and LPS on mitochondrial ROS was not carried out.**

**We have previously reported that 3-AT itself did not induce ROS generation. The drug is a very potent in inhibition of global ROS generation even in the presence of ROS inducers, such as cobalt chloride, in a catalase independent manner [9, 10]. In the present study, 3-AT neither induces global ROS generation (Fig 2A and Fig 4C) nor affects mitochondrial abundance (Fig 1B). Therefore, it is surprising that Walton and Pizzitelli found that 3-AT induces global ROS generation and impairs mitochondrial function [11]. Here, we show that 3-AT did not induce global ROS until 24 h. In addition, 3-AT tends to inhibit the LPS mediated transcriptional activation of pro-inflammatory cytokine genes by 12 h before the evidence for LPS mediated global ROS production at 24 h. Further analysis for the effect of 3-AT on mitochondrial derived ROS was not assessed.**

**Although for a curiosity of reviewer we applied MitoSOX Red superoxide indicator to measure mitochondrial ROS. However, we did not observe any red fluorescence in RAW 264.7 cells treated with LPs alone, 3AT alone, or combination of two chemicals. This result suggests that treatment with 3AT alone, LPS alone, or combination of two chemicals did not induce ROS from mitochondria. We added this content in Discussion**

**Fig R1. ROS from mitochondria were not observable in cells treated with 3-AT alone, LPS alone, or combination of two chemicals.**

(A) Representative red fluorescence image of MitoSOX Red from RAW 264.7 cells treated with 10 mM of 3-AT alone, 100 ng/ml of LPS alone, or combination of both for 24 h . Blue color represents DAPI stained. All images were processed and analyzed in a similar manner

**• Improvements must be made to the statistical analyses. The authors say in the methods that all statistics were performed by two-tailed T-tests, but throughout the manuscript are assessing more than two groups, which should be a one-way ANOVA (as in Figure 1A, D), or two variables (as in Figure 4C, D), which should be a two-way ANOVA, and in both cases statistics should be evaluated with an appropriate post-hoc analysis (Tukey, Holm-Sidak, or Fisher).**

**Response: We agree with the reviewer that if comparisons were made between multiple test groups (comparison between more than two variables) in a single experiment, ANOVA test followed by appropriate post-hoc analysis should be performed. We apologize for the inconveniency faced by reviewer on interpreting our data due to insufficient information supplied in quantified bar graphs. As described in the statistical analysis in Materials and Methods, data obtained from the mean of three independent experiments were shown as ± SD and the differences between experimental values were considered significant at P<0.05 in a two-tailed Student’s-test. We did not perform comparison between multiple test groups and comparisons were only made between two variables (control and test group or between two test groups). To best of our statistical knowledge, Student's t-test is sufficient to interpret our results. Therefore, ANOVA test and post-hoc analysis were not performed. In the revised manuscript, we have illustrated on how comparison between two test groups were made in the quantified bar graph.**

**• Wherever single cell data is produced (eg., Figure 1F, Figure 2B, Figure 2D, etc) the authors should indicate how the fluorescence was quantified (using ImageJ? Some other software?) and more importantly how many cells were quantified by replicate and whether the bars and errors are indicative of the average of single cell data over numerous biological replicates.**

**Response: We have now included detail on how the images were quantified including the software used for measurements in Figure legend and Materials and Methods.**

- **For Fig. 1F, it’s described in Fig 1 legend.**
- **For Fig.2B, it’s described in Materials and Methods under the heading of “measurement of ROS” .**
- **In Fig. 2D, it’s described in Materaials and Methods under the heading of “measurement of Hyper SKL intensity” and highlighted in yellow color.**

**Minor
• More information is needed regarding the Hyper-SKL and HA-ubiquitin plasmids. Source? Backbone? What were the transfection parameters?**

**Response: Hyper-SKL plasmid was gifted by gifted from Dr. Dong-Hyung Cho, Kyung Hee University, South Korea. HA-ubiquitin plasmids (addgene, #18712) already described in Materials and Methods.**

**• What is the specific name of the reverse transcription kit used?**

**Response: Its name is first strand synthesis from Roche (#04379012001) which was included in Materials and Methods.**

**• Please provide specific details on the protease and phosphatase inhibitors used.**

**Response: 1X of protease (GenDEPOT # P3100-001) and phosphatase (GenDEPOT #P3200-001) inhibitor cocktail and inhibitors were added to cell lysis buffers before cell disruption shown in Materials and Methods.**

**• Although it is a common error, the authors are encouraged to use the correct name for the cell line, RAW 264.7. Repeatedly throughout the manuscript, and even in the title, the authors refer to RAW 246.7 cells. A cell line by this name does not exist. See:** [**https://www.atcc.org/products/all/tib-71.aspx**](https://www.atcc.org/products/all/tib-71.aspx)

**Response: We corrected it as RAW 264.7 cells in entire manuscript**

**• Some of the Western Blot images are extremely digitized/pixelated. Wherever possible, the authors are encouraged to include higher resolution images (eg, Supplemental Figure 2, the uncropped blots for Figure 2F – PMP70 and B-actin are inappropriately pixelated).**

**Response: The uncropped western blot results supplied in this study were not overly digitized or pixilated rather we found that there were some problems with the creation of PDF file. In the revised manuscript, we have now included corrected file for all the uncropped western blot results.**

**Additional materials used for the experiments presented in this review**

1. **Mitochondria ROS staining**

Mitochondrial ROS generation was assessed with MitoSOX Red (Invitrogen, M36008). RAW 264.7 cells were treated with 3-AT alone, LPS alone, or combination of both at indicated concentrations and periods, exposed to 1.5 μM MitoSOX Red for 30 min and incubated at 37°C in 5% CO2 incubator. Cells were fixed in 2% paraformaldehyde for 1 h at room temperature, washed, and the coverslips were mounted onto slides. Cells were and then visualized by fluorescence microscopy (IX71; Olympus, Tokyo, Japan). The slides were scored in a blinded manner.

**References**

5. Yang B, Li R, Michael Greenlief C, Fritsche KL, Gu Z, Cui J, Lee JC, Beversdorf DQ, Sun GY. Unveiling anti-oxidative and anti-inflammatory effects of docosahexaenoic acid and its lipid peroxidation product on lipopolysaccharide-stimulated BV-2 microglial cells. J Neuroinflammation. 2018;15(1):202. PMID: 29986724.
6. Lee SB, Kim JJ, Kim TW, Kim BS, Lee MS, Yoo YD. Serum deprivation-induced reactive oxygen species production is mediated by Romo1. Apoptosis. 2010;15(2):204-18. PMID: 19904609.
7. Chen R, Zhu S, Zeng L, Wang Q, Sheng Y, Zhou B, Tang D, Kang R. AGER-Mediated Lipid Peroxidation Drives Caspase-11 Inflammasome Activation in Sepsis. Front Immunol. 2019;10:1904. PMID: 31440260.
8. Choi HB, Ryu JK, Kim SU, McLarnon JG. Modulation of the purinergic P2X7 receptor attenuates lipopolysaccharide-mediated microglial activation and neuronal damage in inflamed brain. J Neurosci. 2007;27(18):4957-68. PMID: 17475804.
9. Lee JN, Kim SG, Lim JY, Dutta RK, Kim SJ, Choe SK. 3‐Aminotriazole protects from CoCl_2_‐induced ototoxicity by inhibiting the generation of reactive oxygen species and proinflammatory cytokines in mice. Arch Toxicol. 2016;90(4):781-91. PMID: 25820916
10. Lee JN, Park J, Kim SG, Kim MS, Lim JY, Choe SK. 3-Aminotriazole protects against cobalt (II) chloride-induced cytotoxicity by inhibiting reactive oxygen species formation and preventing mitochondrial damage in HepG2 cells. Mol Cell Toxicol. 2017;13(1):125-132.
11. Walton PA, Pizzitelli M. Effects of peroxisomal catalase inhibition on mitochondrial function. Front Physiol. 2012; 3:108. PMID: 22536190;
