# Supplementary material for: Pharmacological inhibition of catalase induces peroxisome leakage and suppression of LPS induced inflammatory response in Raw 264.7 cell
Source: PLoS One. 2021 Feb 19;16(2):e0245799. doi: 10.1371/journal.pone.0245799 (PMC7894815; doi:10.1371/journal.pone.0245799)
Supplement: Attachment — Submitted filename: Response to reviewers.docx [file pone.0245799.s014.docx]

**Response to Reviewers**

**Our response**

**We thank PLOS ONE editorial team for valuable comments.**

Reviewer #1: The authors of the revised manuscript **"Pharmacological inhibition of catalase induces peroxisome leakage and suppression of LPS induced inflammatory response in Raw 264.7 cell"** have satisfied all my comments except one.

I asked to demonstrate the interaction between 4HNE and IκBα interaction by Immunofluorescence. The authors argued that was demonstrated by others and that double IP is a better technique to demonstrate interaction than Immunofluorescence (IF). Interaction studies are very difficult, and two techniques should at least demonstrate the same result. The IF would support this interaction. Quite often interactions by IP are in vitro artefact and quite often IP should be demonstrated by mass spectrometry of the bands. An IF would be only quick way to strength the data.

**Response: We appreciate the reviewer's concern regarding the inclusion of additional immunofluorescence (IF) data for showing 4HNE and IκBα interaction.** **We believe that immunoprecipitation (IP) is the standard method to study the interaction of 4HNE with other proteins [1, 2] and IF method could be just an adjunct to standard IP.**

**Although for a curiosity of reviewer we did IF staining in RAW 264.7 cells treated with 3-AT, LPS, or combination of both (Fig S5). IF data clearly suggest the existence of interaction between 4HNE and IκBα in the presence of 3-AT, which is consistent with immunoprecipitation data (Fig 4F). Taken together, we clearly provide the evidence of interaction between 4HNE and IκBα in RAW 264.7 cells.**

**Fig R1. 3-AT promotes the formation of 4HNE-IκBα adduct.**

RAW 264.7 cells pre-treated with 10 mM of 3-AT for 12 h were further incubated with or without 100 ng/ml of LPS for additional 12 h. Cells were then subjected to immunofluorescence with anti-IκBα (green), anti-4-HNE (Red) and DAPI (blue) . Arrow indicate the co-localization of IκBα with 4HNE (yellow).

**Reviewer #2: The authors have sufficiently addressed my concerns and I believe this manuscript is now suitable for publication. However, I would encourage the authors to include the MitoSOX experiment provided in the rebuttal statement as this is relevant data for readers of the manuscript and will contribute to a better understanding of the authors' science and to the field in general.**

**Response: We added the MitoSOX experiment in the main figure (Fig S6).**

**References:**

1. Mihalas BP, De Iuliis GN, Redgrove KA, McLaughlin EA, Nixon B. The lipid peroxidation product 4-hydroxynonenal contributes to oxidative stress-mediated deterioration of the ageing oocyte. Sci Rep. 2017;7(1):6247. PMID: 28740075.
2. Dou X, Li S, Wang Z, et al. Inhibition of NF-κB activation by 4-hydroxynonenal contributes to liver injury in a mouse model of alcoholic liver disease. Am J Pathol. 2012;181(5):1702-1710. PMID: 22982442.
