# Supplementary material for: Pharmacological inhibition of catalase induces peroxisome leakage and suppression of LPS induced inflammatory response in Raw 264.7 cell
Source: PLoS One. 2021 Feb 19;16(2):e0245799. doi: 10.1371/journal.pone.0245799 (PMC7894815; doi:10.1371/journal.pone.0245799)
Supplement: Attachment — Submitted filename: reviewer comments.docx [file pone.0245799.s013.docx]

The authors of the revised manuscript **"Pharmacological inhibition of catalase induces peroxisome leakage and suppression of LPS induced inflammatory response in Raw 264.7 cell"** have satisfied all my comments except one.

I asked to demonstrated the interaction between 4HNE and IκBα interaction by Immunofluorescence. The authors argued that was demonstrated by others and that double IP is a better technique to demonstrate interaction than Immunofluorescence (IF). Interaction studies are very difficult, and two techniques should at least demonstrate the same result. The IF would support this interaction. Quite often interactions by IP are in vitro artefact and quite often IP should be demonstrated by mass spectrometry of the bands. An IF would be only quick way to strength the data.
